# The mediatory role of Majie cataplasm on inflammation of allergic asthma through transcription factors related to Th1 and Th2

**DOI:** 10.1186/s13020-020-00334-w

**Published:** 2020-05-24

**Authors:** Wenting Ji, Qianyi Zhang, Hanfen Shi, Ruijuan Dong, Dongyu Ge, Xin Du, Beida Ren, Xueqian Wang, Qingguo Wang

**Affiliations:** Beijing University of Chinese Medicine, Beijing, 100029 China

**Keywords:** Majie cataplasm, Th2 inflammation of allergic asthma, Th1/Th2, STAT6, GATA-3, STAT3, T-bet

## Abstract

**Background:**

Asthma, a common respiratory disease, is harmful biological effect to our health. As a traditional Chinese medicine for asthma, Majie cataplasm could alleviate the symptoms of asthma and its compositions have immunomodulatory effects. Previous experiments showed that Majie cataplasm was an effective approach to mitigate asthma airway remodeling and had the potential to regulate Th2 cytokines of IL-5 and IL-13. Therefore, our further research focuses on the explanation about the regulatory effect of Majie cataplasm on reshaping Th1/Th2 through their related transcription factors.

**Methods:**

In this experiment, the launch of asthma model was made by inducing with Ovalbumin (OVA) in C57 mice (n = 40), including 4 groups: the untreated control group (n = 10), the asthma model group (n = 10), the dexamethasone group (n = 10) and the Majie cataplasm group (n = 10). After the intervention, all groups of animals got detected for serum IgE levels, and HE staining of lung tissues was to observe and examine pathological changes. Meanwhile, we analyzed the secretion of IL-4^+^ T cells and IFN-γ^+^ T cells in spleen by flow cytometry. The expressions of transcription factor STAT6 mRNA, GATA-3 mRNA and T-bet mRNA in lung tissues was tested by PCR, and western blot had been used to detect levels of JAK2 and STAT3.

**Results:**

We found that Majie cataplasm eased the content of serum IgE and lung inflammation. It could lower the increased number of IL-4^+^ T cells and IFN-γ^+^ T cells (*P *< 0.0001, *P *< 0.01) in asthmatic mice and curb the expression of STAT6 mRNA and GATA-3 (*P *< 0.0001*, P *< 0.01) mRNA as well as the protein levels of JAK2 (*P *< 0.001) and the ratio of pSTAT3/STAT3 (*P *< 0.05). Besides, Majie cataplasm made its mark on T-bet mRNA by improving it (*P *< 0.0001).

**Conclusion:**

These data suggest that Majie cataplasm exert an anti-inflammatory effect of Th2 by rebalancing Th1/Th2 through corresponding transcription factor STAT6, GATA-3, STAT3, and T-bet, which providing a strong cornerstone for asthma control.

## Background

Asthma is one of the most common chronic, non-communicable diseases that threatens people worldwide [1]. Despite the overall decline in asthma mortality rates in adults and children over the past 25 years because of glucocorticoids, still, subsequent problems such as poor compliance and side effects worry us [2]. There is therefore an urgent need for a drug to control the onset of asthma and prevent further deterioration, which involves a complete understanding of the pathogenesis of asthma. Chronic airway inflammation, as the essence of asthma, is the anticipated target for alleviating asthma. As the most common asthma in the clinic, hormone sensitive asthma such as allergic asthma fundamentally stems from high Th2 inflammation [3]. And plenty of literature has shown that T lymphocyte retains influence on asthma. The mainstream view is that the pathogenesis of asthma is the imbalance of T cells differentiation (predominantly Th1/Th2), and various pro-inflammatory factors may greatly assist in Th2 inflammation [4].

Presently, there are different interpretations involving multiple cells for explicating the pathogenesis of asthmatic Th2 inflammation, but mainstream scientists put the blame for the imbalance of T cell differentiation (mainly Th1/Th2). Th cells differentiate into Th1 and Th2 by a certain percentage under healthy conditions, and the two are in a relative balance eliciting robust cellular and humoral immunity. When Th2 differentiation is favored, Th2 cells increase and become functionally hyperactive triggering secretion of proinflammatory factors.

Th2 shift is closely bound up with the upregulation of transcription factor GATA-3 (GATA-3) [5], signal transducer and activator of transcription 6 (STAT6) [6], and signal transducer and activator of transcription 3 (STAT3) [7]. On one hand, they stimulate the proliferation and differentiation of Th2 cells and further promote the secretion of Th2 cytokines such as IL-4, IL-5, and IL-13; on the other hand, they block production of IFN-γ, which in turn aggravates Th2 inflammation [8].

In contrast to Th2 cells, Th1 cells are at a disadvantage in this process. Th1 cells, regulated by the transcription factor t-box transcription factor 21 (T-bet), secrete IL-12, IFN-γ, inhibit Th2 cytokines and are mainly involved in cellular immunity against intracellular pathogens [9]. As Th1 cells and Th2 cells can check and balance each other, the balance between them is the key to alleviate Th2 inflammation.

Majie cataplasm originates from Bai-jie-zi Tufang [10], bringing asthmatic patients a considerable relief. Majie cataplasm contains Ephedra Herba (Mahuang) [11, 12], Semen Sinapis (Baijiezi) [13], Semen Armeniacae Amarum (Kuxingren) [14], Rhizoma Corydalis (Yanhusuo) [15] as well as Rhizoma Zingiberis Recens (ginger) [16, 17], and each of them is able to shape the immune function.

Previous experiments have also found that Majie cataplasm has a regulatory effect on Th2 cytokines like IL-5 and IL-13 [18]. Thus, we speculate that Majie cataplasm is apt to regulate the disequilibrium of Th cell differentiation for asthma treating.

In this study, we discussed the effects of Majie cataplasm on asthmatic mice and orientated the molecular mechanism towards whether it can rebalance Th1/Th2 by regulating the Th2-related GATA-3, STAT6 and STAT3 and Th1-related T-bet.

## Methods

### Mice

6 to 10 week-old WT C57/BL6 mice were purchased from SPF Biotechnology Co., Ltd. (Beijing, China, No. SCXK 2019-0010) and housed indoors under SPF conditions. We conducted this study following the approval of the Ethics Committee on Animal Experiments of the Beijing University of Chinese Medicine (Approval number: No. BUCM-4-2018102401-4015).

### The process of making Majie cataplasm

**Fig. 1:**
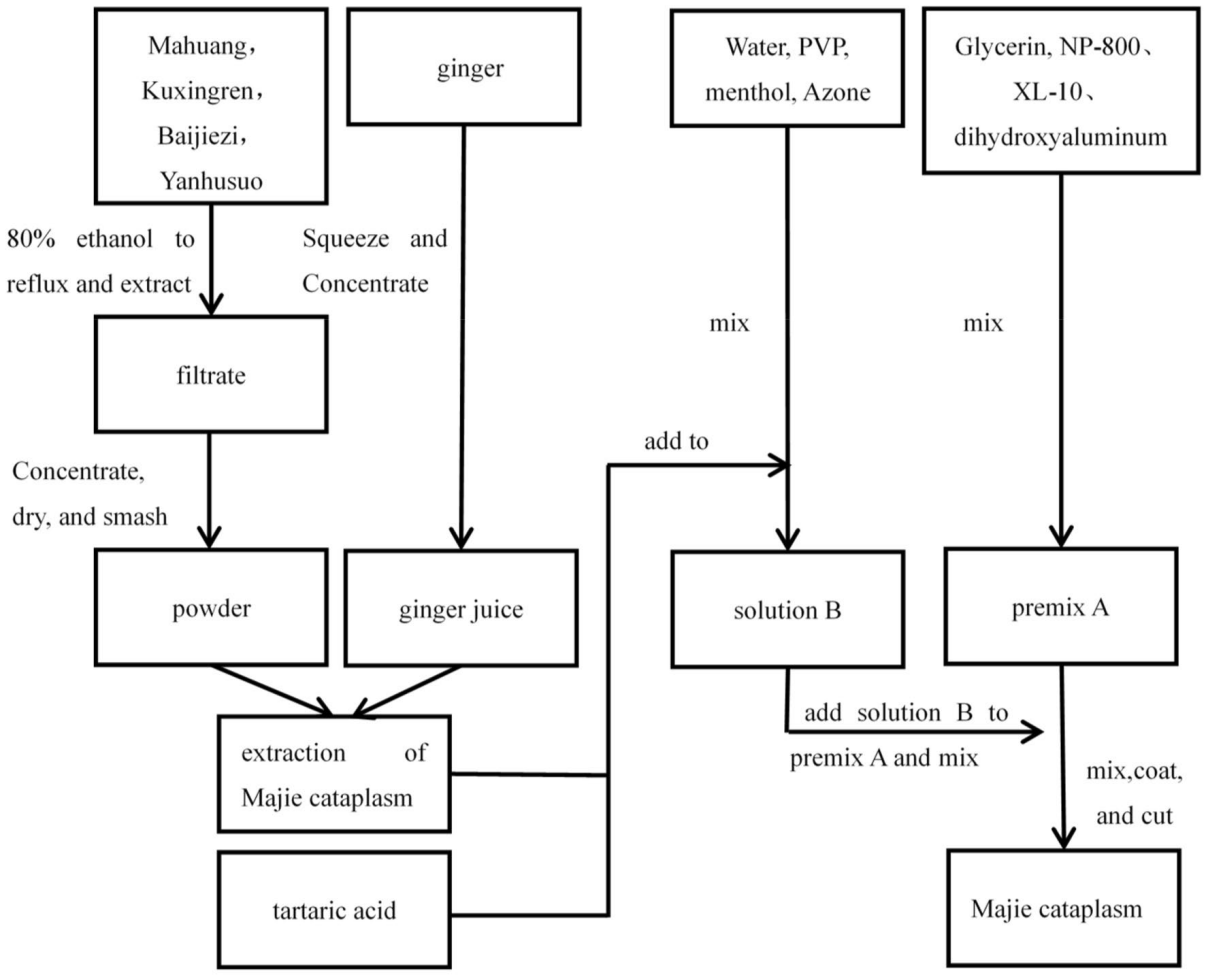
The flow chart of the process for making Majie cataplasm

Except for ginger bought the local Walmart in Beijing, Mahuang, Kuxingren, Baijiezi, and Yanhusuo were purchased from Beijing Tongrentang Pharmaceutical Co. Ltd., China. According our previous experiments, it is dependable to make Majie cataplasm which has good reproducibility and can be mass-produced, which lays a good foundation for the subsequent medicinal mechanism experiments. The fixed dose of these five ingredients of Majie Cataplasm is 4, 4, 4, 4, and 4 g respectively (one piece per day). The contact area of Majie Cataplasm is 63 cm^2^ (length 9 cm × width 7 cm) for humans, which is converted to a mouse application area of about 0.2 cm^2^. Refer to Additional file 1 called “making process of Majie Cataplasm” to see more details. The flow chart of the process for making Majie Cataplasm is as follow in Fig. 1.

### OVA-induced asthma model and the intervention

The OVA-induced asthma model was made as following the method as previously described [19]. On days 0, 7 and 14, C57 mouse (apart from those in the control group) received an intraperitoneal injection (i.p.) about 0.2 mL containing 0.05 mg OVA and 1 mg Alum gel. During days 15–25 the animals were challenged with intranasal dripping of OVA (2.5 mg/ml diluted in PBS, 40 μl/mouse). In contrast, Mice in the untreated control group got injected and challenged with the same amount of PBS.

On the fifteenth day of the experiment, the intervention measures of drugs were as follows: untreated control group (no intervention); asthma model group (no intervention); the dexamethasone group (intraperitoneal injection of dexamethasone sodium phosphate injection 0.2 ml/mouse, 2 mg/kg); the Majie cataplasm group (applied with cataplasm on the mice’s backs, each cataplasm staying for 24 h). All the mice sacrificed after 10 days.

### Histological assessment

The left lung tissues of the mice were taken, dehydrated and fixed with 4% paraformaldehyde, embedded in paraffin, and sectioned 6 μm. Then the sections were performed Hematoxylin-Eosin (H&E) staining.

### Quantitative real-time-PCR (qPCR)

According to instruction, total RNA was collected from lung tissue using HiPure Unviersal RNA Mini Kit (Magen, Shanghai, China), and cDNA was prepared by reverse transcription after dilution with RNA enzyme in water using the cDNA synthesis kit (Thermo Fisher Scientific, Massachusetts, USA). qPCR was performed three times on each sample with SYBR Green reagents (Thermo Fisher Scientific). 50 cycles were monitored on a Step One real-time PCR system (Bio-Rad CFX) for fluorescence intensity quantification. The relative expression of the target genes was calculated by the 2^−Δct^ method and normalized using β-actin. Primers used in this study were as follows (Table 1).

**Table 1:** The primers in the experiment

Primers	Primer sequence
β-acting	Forward: CGTAAAGACCTCTATGCCAA
	Reverse: TTGATCTTCATGGTGCTAGG
GATA-3	Forward: CTCGGCCATTCGTACATGGAA
	Reverse: GGATACCTCTGCACCGTAGC
STAT6	Forward: CACATTTTGGCAGTGGTTTG
	Reverse: CTGGCTCATTGAGGAGAAGG
T-bet	Forward: GGTGTCTGGGAAGCTGAGAG
	Reverse: GAAGGACAGGAATGGGAACA

### Flow cytometry (FCM)

The spleen tissues of mice were finely crushed and filtered before filtrating through a 70 μM filter. The spleen cells were suspended in 1 ml RPMI 1640. Then mouse spleen cells were activated with leukocyte activation cocktail GolgiPlug (BD, New Jersey, USA), 2 µl/10^6^ cells, and incubated for 6 h together. Then cells were washed with PBS containing 1.5% FBS (Thermo Fisher Scientific) and stained with FITC-conjugated anti-CD3 (145-2C11, BD) and PE-Cy7-conjugated CD4 (RM4-5, BD) at 4 °C for 20 min for CD4^+^ T cells.

Thereafter, the cells got incubated in fixation buffer (BD) for 20 min at 4 °C and washed with intracellular staining buffer (BD). Intracellular cytokine staining was performed by using APC-conjugated anti-IFN-γ (XMG1.2, BD), PE-conjugated anti-IL-4 (11B11, BD) in dark for 60 min at room temperature. When cells were washed, they were analyzed by Canto II flow cytometer (BD) and analyzed through FlowJo software (BD).

### Western blot (WB)

The intervention of lung tissue on ice for 10 min with lysate containing 10 mM PMSF for full lysis. The protein concentration of tissue lysates was determined using the BCA Protein Assay Kit (Generay, Shanghai, China). Each lane contains 30 μg of protein and is separated by standard immunoblotting according to standard protocols. Primary antibodies against JAK2 (1:1000), STAT3 (1:1000), pSTAT3 (1:1000) and horseradish peroxidase (HRP)-conjugated secondary antibodies were derived from Cell Signaling Technology, Inc (Boston, USA). Blots were visualized by ECL with GAPDH as the loading control. Western blots were quantified by the FluorChem E system.

### Statistical analysis

Data are expressed as mean ± standard deviation (SD). Differences between groups were analyzed by GraphPad Prism 8 software with one-way ANOVA followed by Bonferroni’s multiple comparison tests. *P *< 0.05 was considered statistically significant.

## Results

### Majie cataplasm could reduce the serum IgE and brought considerable relief to the inflammation in lung

**Fig. 2:**
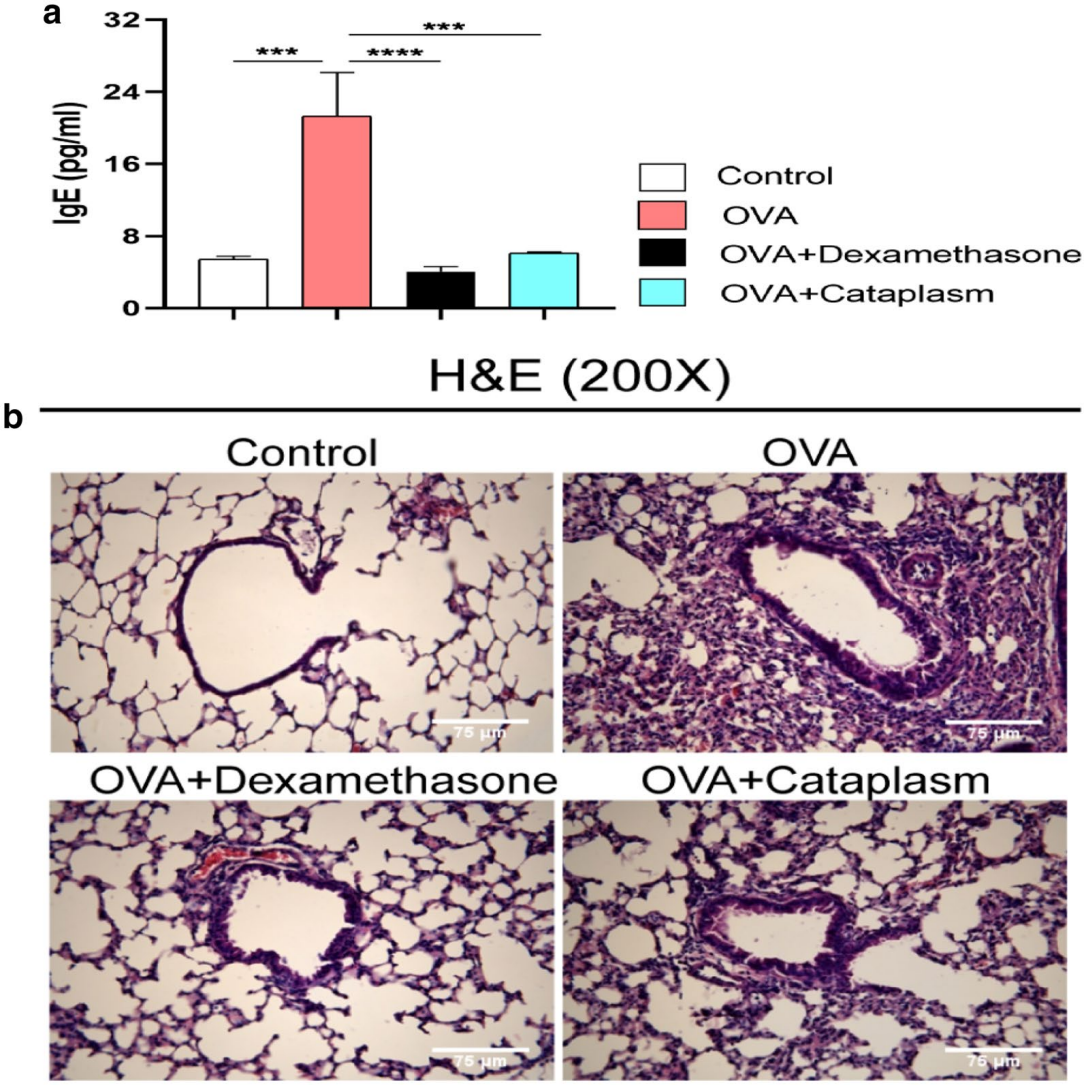
Majie cataplasm alleviated allergic inflammation. **a** The levels of serum IgE was measured by ELISA. **b** H&E stain of lung sections from mice of each group, the pictures are ×200 (scale bars = 75 μm). NS, not significant. *P < 0.05, **P < 0.01, ***P < 0.001 and ****P < 0.0001

We first confirmed the role of Majie cataplasm in the OVA-induced asthma mouse model. The results (Fig. 2a) showed that the allergic inflammation of the asthma model group was significantly higher than that of the control group in terms of the IgE content (*P *< 0.01). Both dexamethasone and Majie cataplasm markedly lowered the content of IgE (*P *< 0.01).

To assess the function of Majie cataplasm in the asthma model, we examined the morphological structure of the lung (Fig. 2b). The control group had normal alveoli, no exudate in the interstitial lung, and few inflammatory cells infiltration. But partial pulmonary consolidation in the asthma model group was observed. With the intravascular congestion and edema, there emerged a large number of inflammatory cells in the alveolar and bronchiole walls. The restored lungs in the dexamethasone group were found with mild thickening of the alveolar wall and a few inflammatory cells in the pulmonary interstitium. After treatment with Majie cataplasm, pneumonia and lesions gradually improved, but the effect is inferior to that in the dexamethasone group. These results indicate that Majie cataplasm can attenuate OVA-induced asthmatic inflammation (Fig. 2).

### The reduction of IL-4^+^ T cells and IFN-γ^+^ T cells was associated with Majie cataplasm

**Fig. 3:**
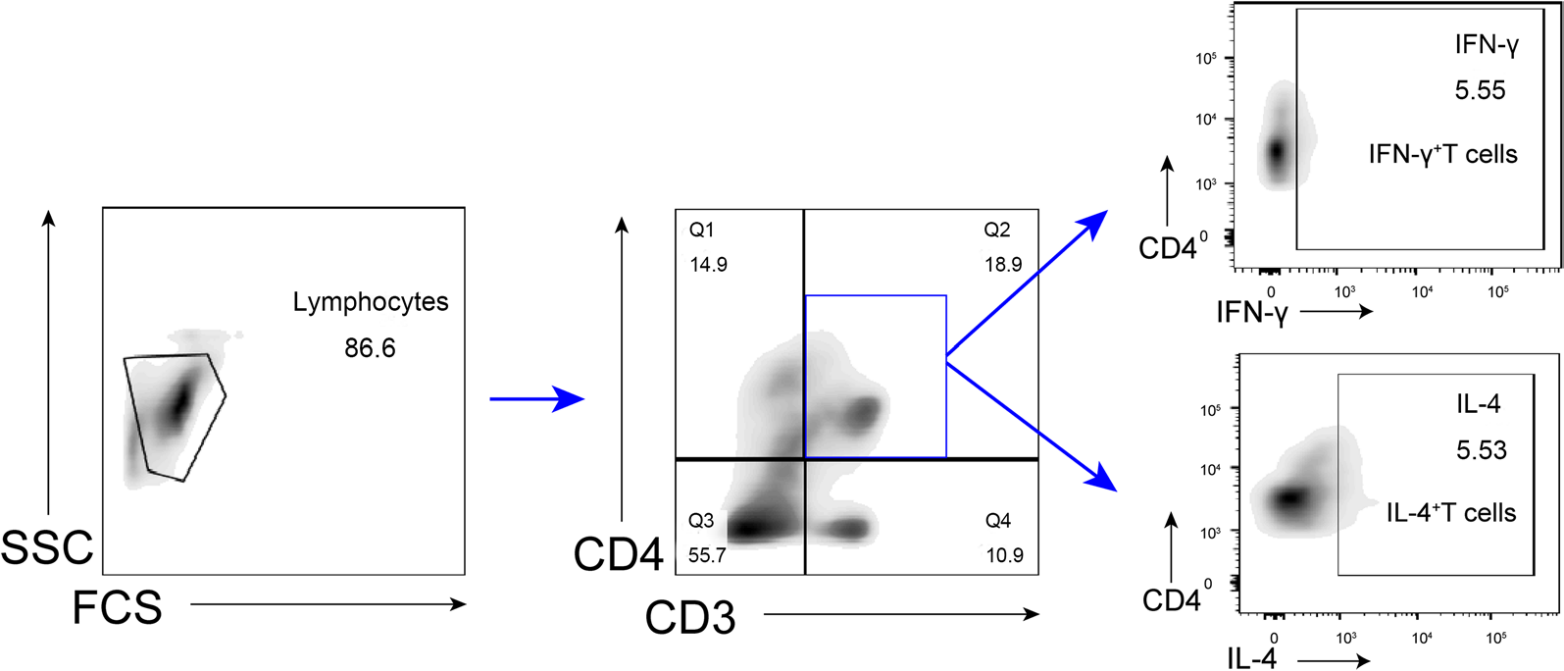
Sequential flow cytometric gating in IFN-γ^+^ T cells and IL-4^+^ T cells in spleen tissue are IFN-γ^+^ T defined as CD3^+^CD4^+^IFN-γ^+^ cells, IL-4^+^ T are defined as CD3^+^CD4^+^IL-4^+^ cells

**Fig. 4:**
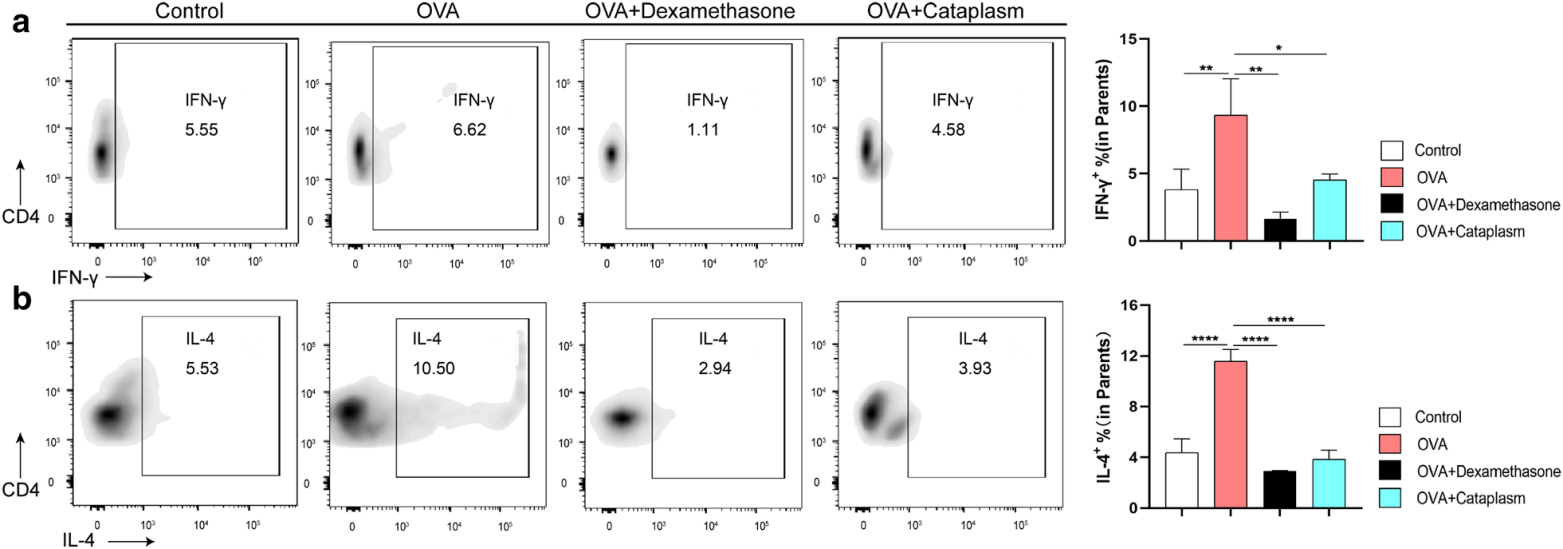
The contents of IL-4^+^ T cells and IFN-γ^+^ T cells. **a** Numbers in outlined areas indicate the percent of spleen IFN-γ^+^ T cells in each group. **b** Numbers in outlined areas indicate percent of spleen IL-4^+^ T cells in each group. NS, not significant. *P < 0.05, **P < 0.01, ***P < 0.001 and ****P < 0.0001

We then checked IL-4^+^ T cells and IFN-γ^+^ T cells from mouse spleen T cells. As shown in Fig. 3, IL-4^+^ T cells and IFN-γ^+^ T cells were circled following the sequential flow cytometric gating strategy. The results of Fig. 4 illustrated the numbers of IL-4^+^ T cells and IFN-γ^+^ T cells in each group. IFN-γ and IL-4 are the representative cytokines secreted by Th1 cells and Th2 cells, respectively. As the numbers of IL-4^+^ T cells and IFN-γ^+^ T cells in spleen tissues can reflect the levels of IFN-γ and IL-4 to a certain extent, we detect IL-4^+^ T cells and IFN-γ^+^ T cells in spleen tissues of each group by FCM. The result showed that the number of IL-4^+^ T cells and IFN-γ^+^ T cells in the asthma model group was significantly higher than that in the control group (*P *< 0.0001, *P *< 0.01).

After intervention with dexamethasone, the levels of IL-4^+^ T cells and IFN-γ^+^ T cells dropped off (*P *< 0.0001, *P *< 0.01). Majie cataplasm had the similar effect (*P *< 0.0001, *P *< 0.05), but the inhibition of IFN-γ^+^ T was weaker than dexamethasone.

### Majie cataplasm had inhibitory effects on the expression of STAT6 mRNA, GATA-3 mRNA and T-bet mRNA

**Fig. 5:**
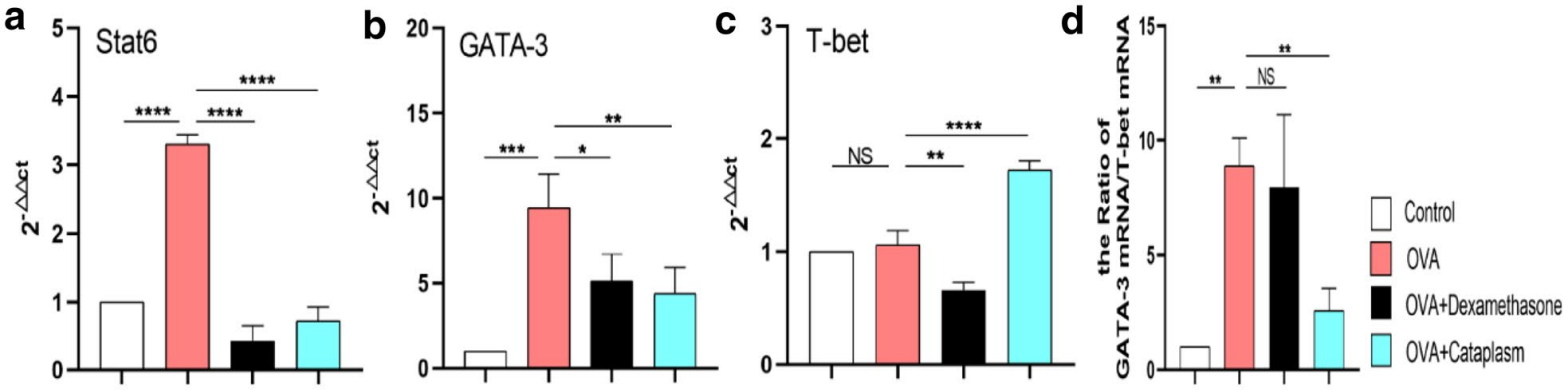
The expressions of STAT6 mRNA, GATA-3 mRNA and T-bet mRNA. STAT6 mRNA and GATA-3 mRNA stood for Th2 and T-bet mRNA represented Th1. **a** The expression of STAT6 mRNA in mouse lungs. **b** The expression of GATA-3 mRNA in mouse lungs. **c** The expression of T-bet mRNA in mouse lungs. **d** The ratio of GATA-3 mRNA/T-bet mRNA. Data are expressed as means of gene expression fold changes relative to β-actin. NS, not significant. *P < 0.05, **P < 0.01, ***P < 0.001 and ****P < 0.0001

The transcription factor STAT6 and GATA-3 closely coordinate their regulation of Th2 differentiation, while the transcription factor T-bet regulates Th1 differentiation. In order to further detect the Th1 and Th2 differentiation in the lung tissues, we then detected the expressions of STAT6 mRNA GATA-3 mRNA and T-bet mRNA. As shown in Fig. 5, the expressions of STAT6 mRNA and GATA-3 mRNA increased compared with the control group (*P *< 0.0001, *P *< 0.001), and both dexamethasone and Majie Cataplasm could obstruct the expression of STAT6 mRNA (*P *< 0.0001, *P *< 0.0001) and GATA-3 mRNA (*P *< 0.05, *P *< 0.01). For T-bet mRNA, its expression had little changed between the control group and the asthma model group. The level of T-bet mRNA decreased in the dexamethasone group (*P *< 0.01), while increased in the Majie Cataplasm group (*P *< 0.0001). Comparing the ratio of GATA-3 mRNA/INF-γ mRNA in each group, it revealed that the ratio of GATA-3 mRNA/INF-γ mRNA in the asthma model group was markedly higher than that in the control group (*P *< 0.001), and no appreciable difference was observed between the dexamethasone group and the asthma model group. But Majie cataplasm intervention caused a decrease in this ratio (*P *< 0.01).

### Majie cataplasm caused the inhibition of the expression of JAK2 and STAT3

**Fig. 6:**
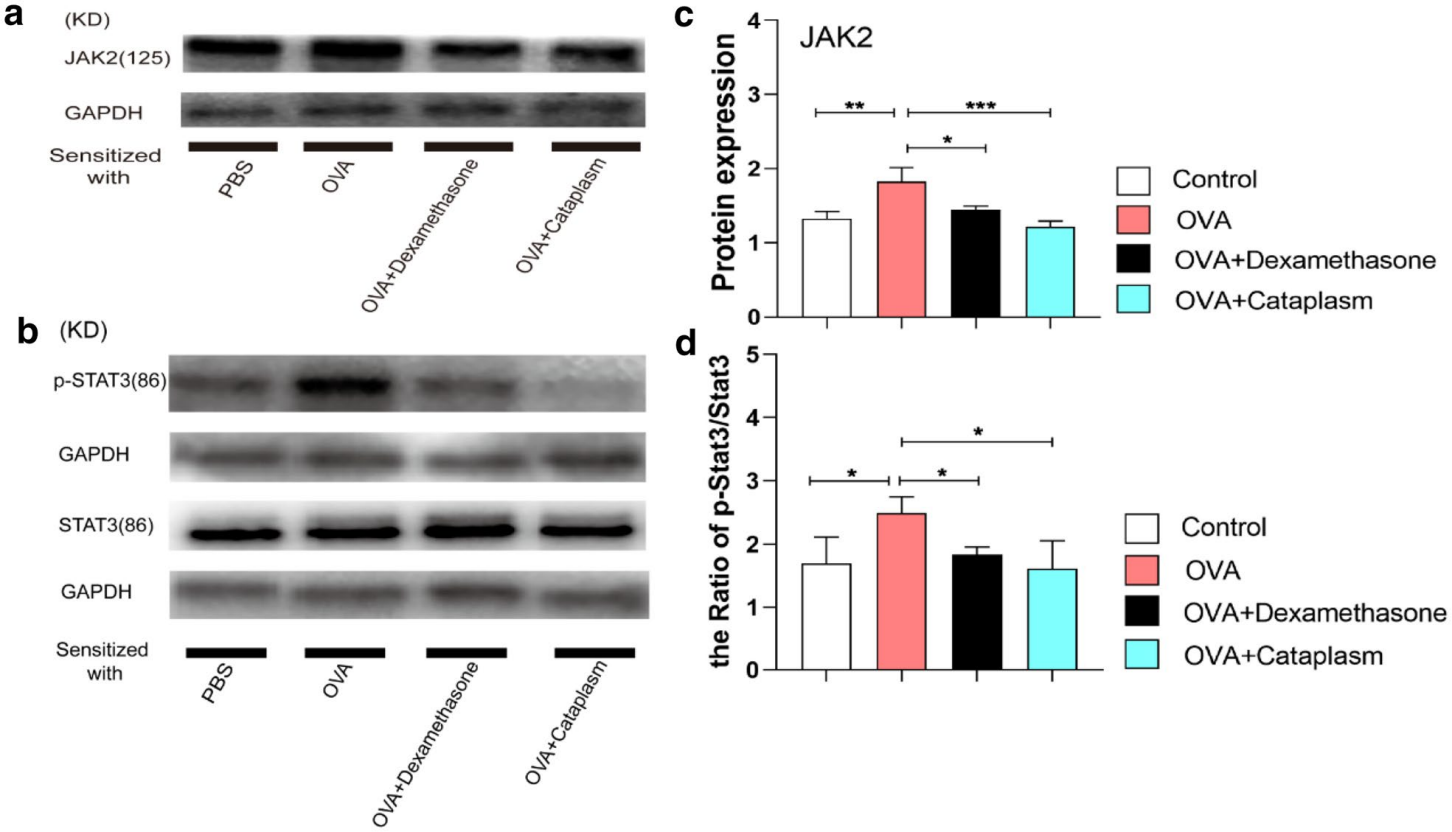
The expressions of JAK2, STAT3 and pSTAT3. **a** Representative western blots showed the expressions of JAK2 in the lungs of each group. **b** The intensity of JAK2 was normalized to GAPDH. **c** Representative western blots showed the expressions of STAT3 and pSTAT3 in the lungs of each group. **d** The intensities of STAT3 and pSTAT3 were normalized to GAPDH, and the ratio of pSTAT3/STAT3 was calculated. NS, not significant. *P < 0.05, **P < 0.01, ***P < 0.001 and ****P < 0.0001

Due to the particularity of transcription factor of STAT3 in Th2 differentiation, we detected STAT3, pSTAT3 and their upstream protein JAK2 by WB. The result in Fig. 6 showed that JAK2 levels rose in the asthma model group (*P *< 0.01), while reduced after the therapeutic intervention of dexamethasone and Majie cataplasm (*P *< 0.05, *P *< 0.001). As for the quantification of STAT3, there was little difference among the four groups, whereas the expression of pSTAT3 varied. The value of pSTAT3/STAT3 in general increased in the asthma model group (*P *< 0.05), while it reduced after the treatment of dexamethasone and Mamie cataplasm (*P *< 0.05, *P *< 0.05).

## Discussion

As one of the most common chronic non-infectious diseases, asthma threatens the health of adults and children. Unfortunately, no standard remedies cure asthma so far. The realistic therapeutic target for asthma is achieving good asthma control by keeping asthmatic inflammation under control.

The pathogenesis of allergic asthma inflammation is mainly based on the imbalances between Th1 and Th2. Under normal circumstances, Th differentiates into Th1 and Th2 according to a certain ratio, and the two are in a relative balance, maintaining the normal cellular immunity and humoral immunity. Once the body is stimulated by abnormal antigens, this balance is tipped. As a result, the function of the Th2 subgroup becomes strengthened, and that of the Th1 subgroup is comparatively weakened, leading to an abnormal immune response [8]. Therefore, restoring the balance of Th1/Th2 is the key to the treatment for asthma. Majie cataplasm, reported effective in asthma treatment, is safe and non-toxic for clinical use [20], and its mechanism has been partly studied [10]. Our previous results enlightened us that Majie Cataplasm could act on Th2 because of its repression on IL-5 and IL-13 belonging to Th2 cytokines. Thus, we focus on the effect of Majie Cataplasm about Th1/Th2. We firstly found that Majie cataplasm reduced the level of serum IgE. At the same time, it alleviated the lung inflammation of asthmatic mice, which lays the groundwork for clinical application. T cells are the main source of IL-4 and IFN-γ which are the representative cytokines about Th1 cells and Th2 cells respectively. Then we detected the numbers of IL-4^+^ T cells and IFN-γ^+^ T cells to roughly speculate Th1 and Th2 differentiation.

The results showed that the number of IL-4^+^ T cells and IFN-γ^+^ T cells increased in the asthma model. Both dexamethasone and Majie Cataplasm had the similar inhibitory effect on IL-4^+^ T cells, while differed on IFN-γ^+^ T cells as Majie Cataplasm did not overly suppress the quantity. Chances are, it regulates the body’s Th1/Th2 shift. We then identified this function by detecting the related transcription factors of Th1 cells and Th2 cells.

Like other members of the signal transduction and activator of transcription (STAT) family, STAT6 has the dual function of signaling molecules and transcription factors. STAT6 is intimately linked to IL-4 and IL-13 exerting a key role in Th2 differentiation. In addition, IL-4/STAT6 is a well-known pathway inducing Th2 differentiation [21–23]. In STAT6 knockout mice, IL-4 stimulation does not induce Th2 related immune responses [24]. Specifically, when IL-4 binds to IL-4 receptor, Stat6 is recruited to IL-4Rα subunit, phosphorylated by tyrosine kinase JAK1 and JAK3, and then transferred to the nucleus, thereby regulating the expression of genes related to Th2 differentiation [25].

In mammals, the transcription factor family of GATA consists of six members, GATA-1 to GATA-6. Among them, GATA-3 is essential for inducing and activating Th2 cells [26]. As a place for T cell development and maturation, in thymus, T cell precursor cells express GATA-3, which can inhibit the potential of B cell development, activate TCR signal transduction, and promote CD4^+^T lineage differentiation after positive selection [27]. While in blood, the level of GATA-3 keeps low in naive T cells, while shoots up after differentiating into Th2 cells. In Th1 cells, GATA-3 barely expresses [28].

GATA-3 can induce and maintain Th2 phenotype in two ways. Firstly, GATA-3 precludes Th1 cell development by down regulating the expression of STAT4 and IL-12 receptor β2 chain [29, 30]. Secondly, GATA-3 can be activated by itself or by other members of GATA family, GATA-1 and GATA-4, and build a positive feedback mechanism to enhance its own expression [31, 32], promote the development of Th2, and obstruct the development of Th1.

Some studies have suggested that GATA-3 could realize its impact on Th2 differentiation through a stronger reliance on STAT6 [33]. STAT6 is involved in the activation of two GATA-3 promoters, thus controlling the activation and maintenance of GATA-3 [34]. Besides, STAT6 is not just engaged in the initiation of Th2 cell differentiation, it also maintains Th2 cell phenotype by inducing the expression of other Th2 related genes and chemokines [35–38]. However, other studies have shown that STAT6^−/−^ mice can still perform Th2 response [39], meaning that GATA-3 could promote Th2 differentiation through other ways like Notch signaling [40] and WNT signaling [41].

Although it has been proved that GATA-3 could be independent in Th2 differentiation, the synergistic effect of STAT6 and GATA-3 stands out. In fact, 26 genes tied with Th2 cells are highly counted on STAT6, and 17 of which could bind to GATA-3 based on DNA chip data [42]. Also, ten genes rely on GATA-3 for transcription, and the remaining 16 genes are transcribed when STAT6 activation form is introduced. So, despite GATA-3 provides a basic for Th2 differentiation, it is inseparable from the function of STAT6. We take them as the staple indicator to evaluate Th2 differentiation and detect them.

T-bet is the essential transcription factors for Th1 cells. T-bet could not only help Th1 development and differentiation, but also block the Th2 differentiation. T-bet blocks Th2 differentiation by directly inhibiting the secretion of IL-4 [43] and preventing the activation of IL-5 and IL-13 through GATA-3 [44]. As a result, Th2 cells increase in T-bet-deficient mice and spontaneously help to form airway hyperresponsiveness associated with overproduction of Th2 cytokines (IL-4, IL-5 and IL-13) and to cause infiltration of eosinophils and lymphocytes [45]. Also, the overexpression of T-bet can increase Th1 cytokines and relieve the proliferation of goblet cells and excessive secretion of mucus in mice after chronic allergen exposure [46]. It can be seen that the expression of T-bet and Th1 cytokines can inhibit asthma Th2 inflammation and airway remodeling.

In order to make further judgment for the differentiation of Th1 and Th2, we detected the expression of STAT6 mRNA, GATA-3 mRNA and T-bet mRNA by qPCR.

The results reveal that dexamethasone and Majie Cataplasm could prohibit the overexpression of STAT6 mRNA and GATA-3 mRNA in the asthma model group, while the effect of the two drugs rarely align for T-bet mRNA. Majie Cataplasm comes to Th1 aid. These results imply that Majie Cataplasm might have additional better adjustment of Th2 shift by boosting Th1 differentiation. Interestingly, the level of T-bet mRNA and IFN-γ^+^ T cells does not reduce in the asthma model, which is likely to be adjusted spontaneously against the increasing number of IL-4^+^ T cells in asthmatic mice.

STAT3 is a member of the family of STAT as well. Activation of STAT3 can promote Th2 differentiation [47], and enhance allergic airway inflammation and airway remodeling [48]. Some laboratory experiments demonstrate that STAT3 is a requisite for the expressions of Th2 related cytokines and transcription factors. As STAT3 and STAT6 match well inTh2 differentiation [49]. Besides, STAT3 is indispensable in Th2 induced B cell-mediated humoral immunity and enhances the conversion rate from IgG1 to IgE [50]. As the upstream of STAT3, Janus kinase 2 (JAK2) can raise STAT3 and make it phosphorylate [51]. We therefore detected the expressions of JAK2, STAT3 and pSTAT3 to explore the effect of Majie Cataplasm on JAK2/STAT3.

WB results suggested that both dexamethasone and Majie Cataplasm could block JAK2/STAT3 pathway as the expression of JAK2 and the ratio of pSTAT3/STAT3 were reduced. And the effect of the two drugs showed little difference. Coupled with the results of the above experiments demonstrate that Majie Cataplasm could help reverse the Th2 shift and reinforce Th1, which is contrary to dexamethasone that although it prevents both Th1 and Th2, the manipulation of Th1/Th2 balance is beyond its capacity. Consequently, Majie Cataplasm may help restore the body’s Th1/Th2 imbalance, yet dexamethasone does not have this potential.

## Conclusion

In conclusion, the inhibitory effect of Majie cataplasm on inflammation of allergic asthma could stem from rebalancing Th1/Th2 through regulating the related transcription factors. We pay attention to the mechanism of Majie cataplasm in the regulation of Th2 inflammation in allergic asthma, especially what differs from corticosteroids. It is not only the bedrock of its efficacy, but also its own edge. As Th2 differentiation comes down to multiple cytokines, cells and neuropeptides, it leaves much to be researched as our study is at the initial stage. Whether Majie Cataplasm can restrain Th2 inflammation through other targets is in progress. In addition, the decryption of this mechanism of Majie cataplasm helps us to understand the multi-target regulation mode of traditional Chinese medicine and to guide its clinical application.

## Supplementary information

**Additional file 1.** Making process of Majie cataplasm.

## Data Availability

The data used to support the findings of this study are available from the corresponding author upon request.

## Abbreviations

JAK2: Janus kinase 2
STAT3: Signal transducer and activator of transcription 3
pSTAT3: Phosphorylated-signal transduction and activator of transcription 3
IL-5: Interleukin 5
IL-13: Interleukin 13
OVA: Ovalbumin
IgE: Immunoglobulin E
H&E: Hematoxylin–Eosin
IL-4: Interleukin 4
IFN-γ: Interferon-γ
GATA-3: Transcription factor GATA binding protein-3
STAT6: Signal transducer and activator of transcription 6
T-bet: Transcription factor T-bet
PBS: Phosphate-buffered saline
PMSF: Phenylmethanesulfonyl fluoride
HRP: Horseradish peroxidase
qPCR: Quantitative real-time-PCR
