# Supplementary material for: The mediatory role of Majie cataplasm on inflammation of allergic asthma through transcription factors related to Th1 and Th2
Source: Chin Med. 2020 May 24;15:53. doi: 10.1186/s13020-020-00334-w (PMC7247251; doi:10.1186/s13020-020-00334-w)
Supplement: Supplementary file 1 — Additional file 1. Making process of Majie cataplasm. [file 13020_2020_334_MOESM1_ESM.docx]

**Part 1. The** **process of** **the extraction of Majie cataplasm**

**1.** **Materials**

Ephedra Herba (Mahuang), Semen Armeniacae Amarum (Kuxingren), Semen Sinapis (Baijiezi), and Rhizoma Corydalis (Yanhusuo) were purchased from Beijing Tongrentang Pharmaceutical Co. Ltd., China. All other reagents were available commercially, including reagents for chromatography.

**2.The method of the extraction of Majie cataplasm**

The medicinal ingredients of Majie cataplasm contain Mahuang, Kuxingren, Baijiezi, Yanhusuo, and Rhizoma Zingiberis Recens (ginger), and the fixed-dose ratio of these five medicines is 1: 1: 1: 1: 1. Except for ginger, which needs to be squeezed and concentrated, the other four drugs should be extracted by a certain method. According to Kong Hui et al. ^[1-2]^, the optimal extraction method was determined. The extraction rate and transdermal rate of pseudoephedrine hydrochloride and amygdalin were comprehensively evaluated. The relevant weight coefficients were 0.25. The data were analyzed by SPSS16.0 software. The experiment arrangement and results are shown in Table 1 and the analysis of variance is demonstrated in Table 2.

Table 1. Orthogonal experiment arrangement and results of extraction process of Majie cataplasm (except for Shengjiang)

| No. | A  Ethanol volume fraction  /% | B  Extraction time/h | C  Extraction times | D  (Blank) | Extraction rate of pseudoephedrine hydrochloride/% | Total transdermal rate of pseudoephedrine hydrochloride/% | Extraction rate of amygdalin/% | Total transdermal rate of amygdalin  /% | The comprehensive score |
| --- | --- | --- | --- | --- | --- | --- | --- | --- | --- |
| 1 | 60 | 0.5 | 1 | 1 | 0.69 | 64.06 | 0.68 | 41.14 | 69.85 |
| 2 | 60 | 1 | 2 | 2 | 1.31 | 38.28 | 1.41 | 22.87 | 72.72 |
| 3 | 60 | 1.5 | 3 | 3 | 1.62 | 31.96 | 1.29 | 17.68 | 69.63 |
| 4 | 70 | 0.5 | 2 | 3 | 1.06 | 54.02 | 0.81 | 32.93 | 69.80 |
| 5 | 70 | 1 | 3 | 1 | 1.41 | 32.11 | 1.35 | 15.15 | 66.46 |
| 6 | 70 | 1.5 | 1 | 2 | 0.96 | 69.98 | 0.89 | 40.27 | 79.33 |
| 7 | 80 | 0.5 | 3 | 2 | 1.38 | 59.06 | 1.07 | 40.92 | 85.75 |
| 8 | 80 | 1 | 1 | 3 | 0.91 | 45.92 | 0.58 | 28.32 | 57.42 |
| 9 | 80 | 1.5 | 2 | 1 | 1.37 | 72.05 | 1.14 | 41.38 | 91.96 |
| *K_1_* | 212.20 | 225.40 | 206.60 | 228.27 |  |  |  |  |  |
| *K_2_* | 215.59 | 196.60 | 234.48 | 237.80 |  |  |  |  |  |
| *K_3_* | 235.13 | 240.92 | 221.84 | 196.85 |  |  |  |  |  |

Table 2. Analysis of variance of comprehensive score

| Soruces of variation | *SS* | *f* | *MS* | *F* | *P* |
| --- | --- | --- | --- | --- | --- |
| A | 102.121 | 2 | 51.06 | 0.334 | >0.05 |
| B | 337.175 | 2 | 168.587 | 1.102 | >0.05 |
| C | 129.925 | 2 | 64.962 | 0.424 | >0.05 |
| D(error) | 306.104 | 2 | 153.052 |  |  |

Note：F0.05(2.2)=19

From results in table 1, it can be seen that the role of three factors-extraction time, extraction times and ethanol volume fraction, weakened in turn. However, they had no significant effect on the extraction process. The best extraction method was determined as A3B3C2, which was to mix equal amounts of Mahuang, Kuxingren, Baijiezi, and Yanhusuo. 6 times of 80% ethanol was added for reflux extraction twice, each time for 1.5h.

Summarizing the experimental results and combining the preparation method of ginger juice, the optimal process of the complete extraction process of Majie cataplasm is as follows:

Take the same amount of 4 flavors except for ginger, then add 6 times the amount of 80% ethanol to reflux and extract 2 times, each time for 1.5 hours.

Ethanol is recovered through a vacuum at 60℃ and the remainder is dried under reduced pressure (-0.08MPa, 60℃). The dry remainder is smashed to powder and reserved properly (the yield of dry extraction is about 10%). Take the ginger according to the prescription proportion，it is squeezed to obtain the juice, which is then centrifuged at 8000×g for 5 min at room temperature. The supernatant is taken and concentrated at 60℃ under reduced pressure to obtain the concentrated ginger juice and saved at 4℃ until use. The powder is mixed with ginger juice according to the prescription proportion (1: 1: 1: 1: 1), which is as the extraction of Majie cataplasm for the subsequent HPLC test.

**3. The detection of ephedrine hydrochloride, pseudoephedrine hydrochloride and amygdalin in the extraction of Majie cataplasm**

According to the Chinese Pharmacopoeia (2010 edition) and analysis of Majie cataplasm, Mahuang is a crucial drug that affects very strongly on the treatment of asthma, and Kuxingren and Baijiezi are used as auxiliaries. Thus, the principal components of these drugs could be considered as evaluation indexes for Majie cataplasm. It is worth noting that Baijiezi could stimulate the skin, increase the temperature of the epidermis, and promote the absorption of drugs ^[2]^. Therefore, it is mostly as an important indicator of evaluating topical drugs like this kind, and we tested it in the final product of Majie cataplasm. For the detection of the extraction of Majie Cataplasm, ephedrine hydrochloride, pseudoephedrine hydrochloride and amygdalin were selected.

**3.1 The detection of ephedrine hydrochloride and pseudoephedrine hydrochloride in the extraction of Majie cataplasm**

**Materials**

Waters 1525 high performance liquid chromatograph (including 1525 Binary HPLC Pump, 2487 Dual register Absorbance Detector, 2707 Autosampler). Ephedrine hydrochloride and pseudoephedrine hydrochloride (National Institute for the Control of Pharmaceutical and Biological Products, Beijing, batch No.171241-201007 and No.171237-201208).

**HPLC Conditions**

A Waters XTerra RP_18_5 μm (4.6 mm x 250 mm) column was used for chromatography at a column temperature of 25°C. The mobile phase consisted of acetonitrile-0.1% phosphoric acid solution (4:96). The flow rate was set at 1.0ml/min and the detection wavelength was 210 nm. The injection volume was 10μl.

**Solution Preparation**

**Preparation of the Standard Solution**

The appropriate amount of ephedrine hydrochloride and pseudoephedrine hydrochloride was precisely weighed, and the two standards were dissolved in methanol to obtain the mixture solution at a concentration of 0.05mg·ml^−1^, respectively.

**Preparation of the Sample Solutions**

Took approximately 0.2g of the extraction of Majie cataplasm. Then it was poured into a 100ml volumetric flask, diluted with methanol to the mark, and filtered (0.45μm microporous filter membrane). Then got the filtrate.

**Preparation of the solution of Mahuang Extraction**

Took approximately 0.5g of raw Ephedra powder (passed through No. 2 sieve), and

weighed it accurately. Then it was poured into a stoppered conical flask, added accurately 50ml of 1.44% phosphoric acid solution. Weighed and sonicated (power 600W, frequency 50kHz) 20 minutes. Weighed again after cooling, and made up the lost weight with a 1.44% phosphoric acid solution. Then shook completely, filtered, and got the filtrate.

**Detection**

The accurate injection volume of the standard solutions, the sample solutions and the Mahuang extraction was 10μl, respectively. Then injected into the HPLC analyzer for detection. The results were shown in the following tables.

Table 3. The content of ephedrine hydrochloride in the standard solutions, the sample solutions and the Mahuang extraction

| Sample | Weight (mg) | Sample concentration  (mg/ml) | Peak area | Average peak area | Measured concentration  (mg/ml) | Percentage content  (%) | Average content  (%) | Transfer rate  (%) |
| --- | --- | --- | --- | --- | --- | --- | --- | --- |
| Standard | 1.3 | 0.052 | 1231614 | 1231596.5 |  |  |  |  |
|  |  |  | 1231579 |  |  |  |  |  |
| Extraction 1 | 203.1 | 2.031 | 895157 | 878101.0 | 0.07 | 3.65 | 3.64 | 67.46 |
|  |  |  | 861045 |  |  |  |  |  |
| Extraction 2 | 200.0 | 2.000 | 858287 | 858724.5 | 0.07 | 3.63 |  |  |
|  |  |  | 859162 |  |  |  |  |  |
| Mahuang-1 | 500.3 | 10.006 | 3226602 | 3198579.5 | 0.135 | 1.35 | 1.35 |  |
|  |  |  | 3170557 |  |  |  |  |  |
| Mahuang-2 | 500.6 | 10.012 | 3249926 | 3193929.5 | 0.135 | 1.35 |  |  |
|  |  |  | 3137933 |  |  |  |  |  |

Note: Standard was from Standard Solution, Extraction 1 and Extraction 2 were from the solution of the extraction of Majie Cataplasm and Mahuang-1 and Mahuang-2 were from the solution of Mahuang extraction.

Table 4. The content of pseudoephedrine hydrochloride in the standard solutions, the sample solutions and the Mahuang extraction

| Sample | Weight (mg) | Sample concentration  (mg/ml) | Peak area | Average peak area | Measured concentration  (mg/ml) | Percentage content  (%) | Average content  (%) | Transfer rate (%) |
| --- | --- | --- | --- | --- | --- | --- | --- | --- |
| Standard | 1.3 | 0.052 | 1111351 | 1090862.0 |  |  |  |  |
|  |  |  | 1070373 |  |  |  |  |  |
| Extraction 1 | 203.1 | 2.031 | 484783 | 482989.5 | 0.05 | 2.27 | 2.23 | 49.74 |
|  |  |  | 481196 |  |  |  |  |  |
| Extraction 2 | 200.0 | 2.000 | 459309 | 459354.0 | 0.04 | 2.19 |  |  |
|  |  |  | 459399 |  |  |  |  |  |
| Mahuang-1 | 500.3 | 10.006 | 2369172 | 2344705.5 | 0.112 | 1.12 | 1.12 |  |
|  |  |  | 2320239 |  |  |  |  |  |
| Mahuang-2 | 500.6 | 10.012 | 2387912 | 2358431.5 | 0.112 | 1.12 |  |  |
|  |  |  | 2328951 |  |  |  |  |  |

Note: Standard was from Standard Solution, Extraction 1 and Extraction 2 were from the solution of the extraction of Majie Cataplasm and Mahuang-1 and Mahuang-2 were from the solution of Mahuang extraction.

**3.2 Detection of amygdalin in the extraction of Majie cataplasm**

**Materials**

High performance liquid chromatograph: Agilent 1100 series, including online degasser: G1379A DEGASSER. Chromatographic pump: Agilent G1311AQuat pump. Column temperature box: G1316A COLCOM. Sample injector: Agilent G1329A ALS. Detector: Agilent G1315B DAD. Chromatographic workstation: Agilent Chemistation for LC system. Amygdalin (National Institute for the Control of Pharmaceutical and Biological Products, Beijing, batch No. 110820-201004)

**HPLC Conditions**

Column: Agilent ZORBAX ODS 5 μm (4.6 mm x 250 mm), mobile phase: acetonitrile-0.1% phosphoric acid solution (8:92), column temperature: 25℃, flow rate: 1.0ml·min-1, detection wavelength: 207nm.

**Solution Preparation**

**Preparation of the Standard Solution**

The appropriate amount of amygdalin standard was dissolved in methanol to obtain the solution at a concentration of 0.30mg·ml^−1^.

**Preparation of the Sample Solution**

Took approximately 1.0g of extraction, and weighed it accurately. Then it was poured into a 100ml volumetric flask, diluted with 80% methanol to the mark, and filtered (0.45μm microporous filter membrane). Then got the filtrate.

**Preparation of the solution of Kuxingren Extraction**

Took approximately 0.25g of Kuxingren powder (passed through No. 2 sieve), and weighed it accurately. Then it was poured into a stoppered conical flask, added accurately 25ml methanol. Weighed and sonicated (power 250W, frequency 50kHz) for 30 minutes. Weighed again after cooling, and made up the lost weight with methanol. Then shook and filtered. Precisely measured 5ml of the filtrate, and it was poured into a 50ml volumetric flask, and diluted with 50% methanol to the mark. Shook and filtered (0.45μm microporous filter membrane), then got the filtrate.

**Detection**

The accurate injection volume of the standard solutions and the Kuxingren extraction was 10μl, respectively. The sample solution was 5μl. Then injected into the HPLC analyzer for testing. The results are shown in the following table.

Table 5. The content of amygdalin in the standard solutions, the sample solutions and the Kuxingren extraction

| Sample | Weight  (mg) | Sample concentration  (mg/ml) | Peak area | Average peak area | Measured concentration  (mg/ml) | Percentage content  (%) | Average content  (%) | Transfer rate  (%) |
| --- | --- | --- | --- | --- | --- | --- | --- | --- |
| Standard | 2.99 | 0.28 | 2434.8 | 2463.9 |  |  |  |  |
|  |  |  | 2492.9 |  |  |  |  |  |
| Extraction | 986.0 | 9.86 | 2739.8 | 2773.8 | 0.63 | 6.39 |  | 52.39 |
|  |  |  | 2807.7 |  |  |  |  |  |
| Kuxingren-1 | 255.3 | 1.021 | 282.8 | 282.3 | 0.032 | 3.14 | 3.05 |  |
|  |  |  | 281.7 |  |  |  |  |  |
| Kuxingren-2 | 255.9 | 1.024 | 265.9 | 266.7 | 0.030 | 2.96 |  |  |
|  |  |  | 267.5 |  |  |  |  |  |

Note: Standard was from Standard Solution, Extraction 1 and Extraction 2 were from the solution of the extraction of Majie Cataplasm and Kuxingren-1 and Kuxingren-2 were from the solution of Kuxingren extraction.

1. **Conclusion**

The results showed that by this extraction method, the contents of ephedrine hydrochloride, pseudoephedrine hydrochloride, and amygdalin in the extraction were well measured respectively. The extraction rate of Majie cataplasm was 10%. The content of ephedrine hydrochloride was 3.64%, pseudoephedrine hydrochloride was 2.23%, and amygdalin was 6.39%, the transfer rate of ephedrine hydrochloride was 67.46%, pseudoephedrine hydrochloride was 49.74%, and for amygdalin was 52.39%, The extraction method can extract the main chemical components, and the process conditions are reasonable.

**Part 2.** **Molding technique of Majie cataplasm**

The single-factor experimental method was used to explore the optimum molding process of Majie cataplasm. The results are as follows.

Firstly, take 2.0kg glycerin, add 0.50kg NP-800, 0.12kg XL-10, and 0.042kg dihydroxyaluminum, and stir to allow the three polymer materials to be fully mixed in the glycerol to make a polymer glycerol premix A. Secondly, add 0.14kg PVP to 4.5kg water to dissolve, then add 0.0475kg Azone and 0.0475kg menthol, mix them well and add 1.65kg the dry extraction powder of Majie cataplasm with 850ml ginger concentrate and 0.02kg tartaric acid as solution B. Thirdly, add solution B to premix A and stir. After stirring and mixing thoroughly, the final mixture is coated, cut, and packed. The contact area of each piece of cataplasm is 63cm^2^ (9.0cm in length, 7.0cm in width), and it weighs about 7.0g. Because Majie cataplasm belongs the category of gel paste, we refer to the standard of Chinese pharmacopoeia (2010 edition) and our cataplasm meet the needs.

Ointment content: according to the general rule of the fourth part of “Chinese pharmacopoeia” in 2010 edition, each 100cm^2^ should be no less than 7.0g.

Adhesiveness: according to the first method of 0952 general rule of the fourth part of “Chinese pharmacopoeia” 2010 edition, it should be able to adhere to the No. 3 steel ball.

Formability: according to the 0122 general rule of the fourth part of “Chinese pharmacopoeia” in 2010 edition, there should be no flowing phenomenon on the cataplasm surface.

Microbial limits: based on Non-sterile product microbial limiting inspection including microbiological counting method (General Rule 1105), control bacteria inspection method (General Rule 1106) and non-sterile drug microbial limiting standards (General Rule 1107) inspection, Majie cataplasm meets all the requirements. (the general rule of the fourth part of “Chinese pharmacopoeia” in 2010 edition).

For details of this part, please refer to the patent related to Majie cataplasm (CN104174007A).

**Part 3. Thin-layer identification of Majie cataplasm**

In line with the “Chinese pharmacopoeia” (2010 edition), all five medicinal materials including Mahuang, Kuxingren, Baijiezi, Yanhusuo, and ginger in the prescription of Majie cataplasm were selected for thin-layer identification research. During the experiment, the preparation method of Kuxingren samples was very complicated, and the result of Baijiezi was unstable though the identification was carried out by multiple development systems and multiple samples preparation methods. Therefore, Mahuang, Yanhusuo, and ginger was finally selected as the identification items of Majie cataplasm by thin-layer chromatography method.

**1. The thin-layer identification of Mahuang**

**1.1 Solution preparation**

**1.1.1 Preparation of the sample solution**

Took 2 pieces of this product, removed the coating, cut into 0.3cm square pieces, added a few drops of concentrated ammonia solution, the added 10ml of chloroform, heated and refluxed for 1 hour. Then filtered and concentrated the filtrate to 1ml as the sample solutions.

**1.1.2 Preparation of the standard solution**

Took ephedrine hydrochloride standard and added methanol to make a solution containing 1.0mg per 1ml as the standard solution.

**1.1.3 Preparation of the Mahuang(crude drug) solution**

Took 0.5g of Mahuang and added a few drops of concentrated ammonia, added 10ml chloroform, heated and refluxed for 1 hour. Then filtered and the filtrate was concentrated to 1ml as the ephedra solutions.

**1.1.4 Preparation of the negative solution**

Took drugs except for Mahuang in compliance with the prescription of Majie cataplasm, handled it under the process of the extraction and the molding technique of Majie cataplasm, and then made the negative sample solution under the method of the sample solution above.

**1.2 Detection**

According to thin-layer of "Mahuang" section [identification] in the first part of “Chinese pharmacopoeia” in 2010 edition, took 5μl from the above solutions respectively, pointed them on the same silica gel G thin-layer plate. Chloroform-methanol-concentrated ammonia (20:2.5:0.3) was as the developing agent. Then sprayed with ninhydrin solution at 105℃ until the spots became clear. The result illustrated that the same red spots appeared at the corresponding positions of the chromatogram of the ephedra solutions, the standard solutions and the sample solutions.


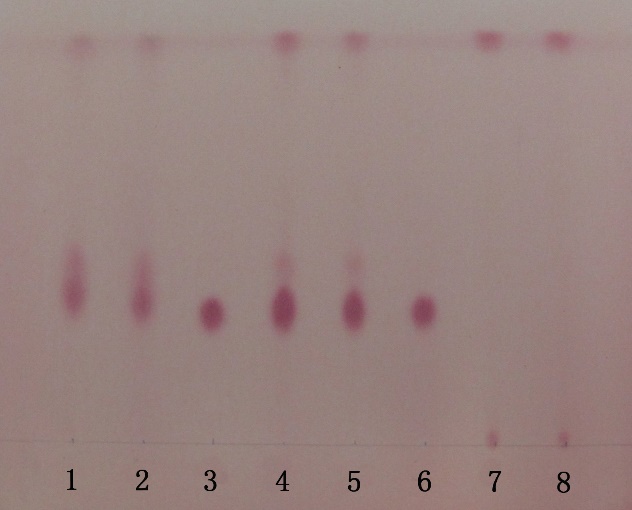


Fig 1 TLC of Mahuang

1, 2 from the Mahuang (crude drug) solution 3, 6 from the standard solution 4, 5from the sample solution 7, 8 from the negative solution

**2. The thin-layer identification of Yanhusuo (Rhizoma Corydalis Yanhusuo)**

**2.1 Solution preparation**

**2.1.1 Preparation of the sample solutions**

Took 2 pieces of this product, removed the coating, and cut into 0.3cm square pieces. Added 50ml methanol, sonicated for 30min, filtered and evaporated the filtrate to dry. Then added 10ml water to dissolve, and added concentrated ammonia to make the solution alkaline. Then it was extracted 3 times with ether (10ml per time). The extraction combined and evaporated to dry. The residue was dissolved by adding 1ml of methanol as the sample solution.

**2.1.2 Preparation of the standard solution**

Took the tetrahydropalmatine and added methanol to make a solution containing 0.5mg per 1ml as the standard solution.

**2.1.3 Preparation of** **the Yanhusuo (crude drug) solution**

Took 1.0g of Yanhusuo, add 50ml methanol, sonicated for 30min. After filtration, the filtrate was evaporated and dried, which was dissolved with 10mlwater. Concentrated ammonia was added and adjusted to alkalinity. Then it was extracted 3 times with 10ml of ether each time. The extraction combined and evaporated to dry, and the residue was dissolved in 1ml of methanol as a Yanhusuo (crude drug) solution.

**2.1.4** **Preparation of the negative solution**

Took drugs except for Yanhusuo according to the prescription of Majie cataplasm, handled it under the molding technique of Majie cataplasm, and then made the negative sample solution following the reference of the method of the sample solution.

**2.2 Detection**

According to thin-layer of "Yanhusuo" section [identification] in the first part of “Chinese pharmacopoeia” in 2010 edition, took 10μl from the sample solution, 5μl from the remaining solutions respectively, pointed them on the same silica gel G thin-layer plate, and used toluene-acetone (6∶2) as a developing agent. Then smoked with iodine vapor until the spots were clear. Evaporated the iodine, and observed under the UV lamp (365nm). The result showed that the same color spots appeared at the corresponding positions of the chromatogram of the Yanhusuo (crude drug) solution, the standard solution, and the sample solutions.


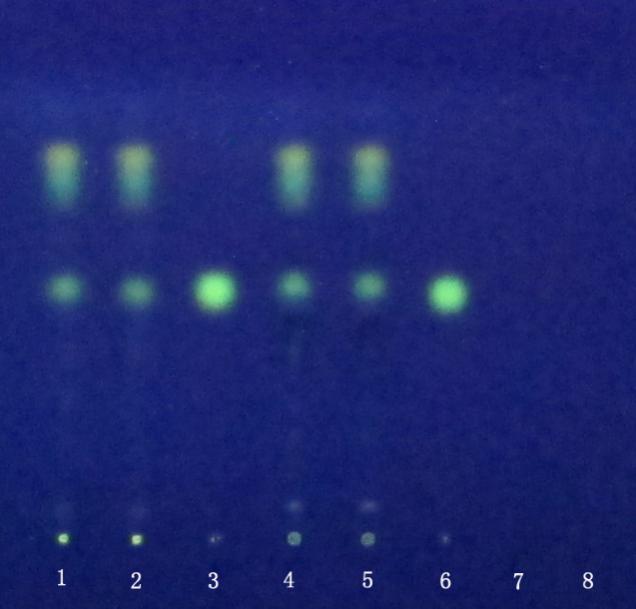


Fig 2 TLC of Yanhusuo

1, 2 from the Yanhusuo (crude drug) solution 3, 6 from the standard solution

4, 5 from the sample solutions 7, 8 from the negative solution

**3. The thin-layer identification of Ginger**

**3.1 Solution preparation**

**3.1.1 Preparation of the sample solution**

Took 2 pieces of this product, removed the coating, cut into 0.3cm square pieces, add 20ml ethyl acetate and sonicated for 10 minutes. Then filtered, evaporated the filtrate, and added 1ml ethyl acetate to dissolve the residue.

**3.1.2 Preparation of the standard solution**

Took 6-gingerol and add ethyl acetate to make a solution containing 0.5mg per 1ml.

**3.1.3 Preparation of the negative solution**

Other drugs except ginger were taken as the prescription of Majie cataplasm. It was handled under the molding technique of Majie cataplasm, and then made the negative sample solution under the method of the sample solution.

**3.2 Detection**

According to thin-layer of "Ginger" section [identification] in the first part of “Chinese pharmacopoeia” in 2010 edition, took 5μl from the above solutions respectively, pointed them on the same silica gel G thin-layer plate. Petroleum ether (60~90℃)-trichloromethane-ethyl acetate (5:2:3) was used as a developing agent. Then sprayed with vanillin sulfuric acid solution at 105 °C until the spots became clear. The result showed that the spots of the same color appeared at the corresponding positions of the chromatogram of the sample solution and the standard solution.


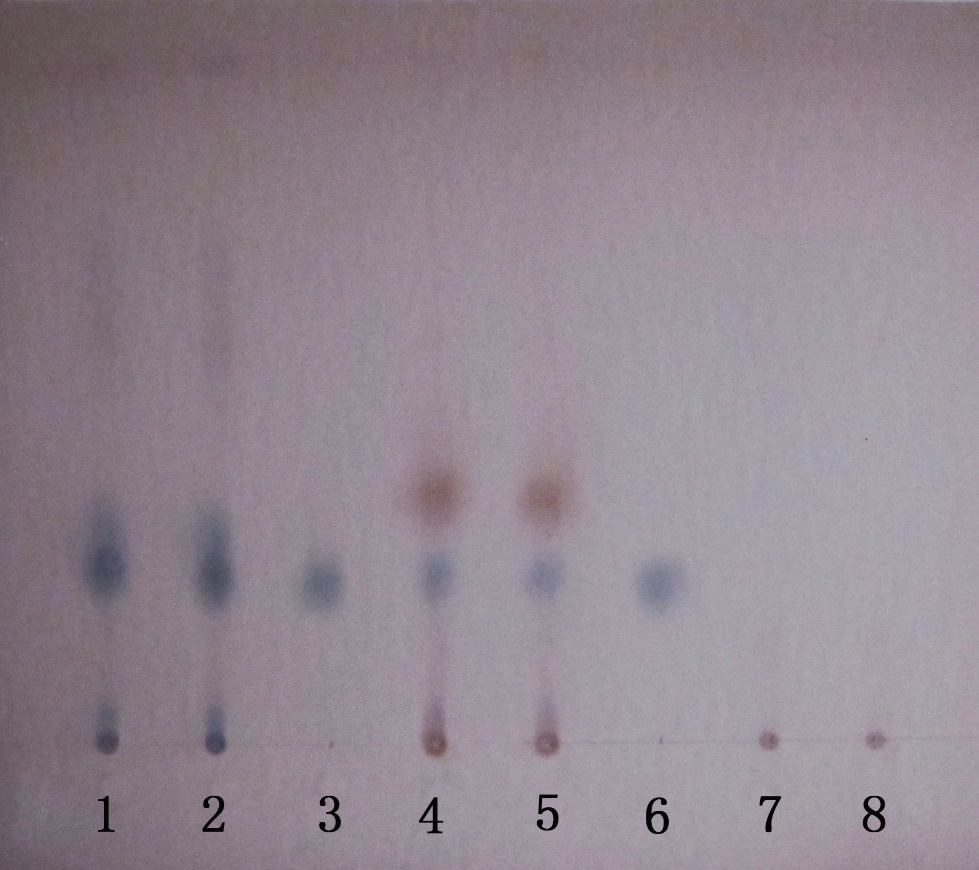


1 2 3 4 5 6

Fig 3 TLC of Ginger

1, 4 from the standard solution; 2, 3 from the sample solution; 5, 6 from the negative solution

**Part4. Determination of ephedrine hydrochloride, pseudoephedrine hydrochloride and sinapine thiocyanate in Majie cataplasm by HPLC**

Majie cataplasm is the external treatment for asthma. Mahuang is one of the drugs that fulfill a major role. Its main ingredients, ephedrine hydrochloride and pseudoephedrine hydrochloride, are stable and the HPLC detection method is feasible. In addition, Baijiezi has its unique advantages in external use. Therefore, ephedrine hydrochloride, pseudoephedrine hydrochloride in Mahuang, and sinapine thiocyanate in Baijiezi are selected as the indicative components of Majie cataplasm.

**1.** **Materials**

Instruments: American Agilent1100, Agilent1260, waterse2698 high performance liquid chromatography, quaternary pump, DAD detector, autosampler, online vacuum degasser, intelligent column oven and Agilent ChemStation, SK8200HP ultrasonic cleaning instrument, -1001 rotary evaporator, AG285 electronic balance.

Materials: Yunnan Baiyao Group Co., Ltd. provides 4 batches of final product according to the process of the extraction and the molding technique of Majie cataplasm, the batch numbers are: 20130901, 20131101, 20131201 and 20131202.

Ephedrine hydrochloride standard (provided by National Institute for the Control of Pharmaceutical and Biological Products, Beijing, batch number: 171241-201007, purity: 99.7%), pseudoephedrine hydrochloride standard (provided by National Institute for the Control of Pharmaceutical and Biological Products, Beijing, batch number: 171237-201208, purity: 99.9%). Negative samples of Majie cataplasm lacking Mahuang shall be prepared in line with the process of the extraction and the molding technique of Majie cataplasm. Sinapine thiocyanate standard (provided by National Institute for the Control of Pharmaceutical and Biological Products, Beijing, batch number: 111702-201102, purity: 98.2%). Negative samples, Baijiezi deficiency of Majie cataplasm, were prepared according to the process of the extraction and the molding technique of Majie cataplasm.

The mobile phase, methanol and acetonitrile, is chromatographically pure. Potassium dihydrogen phosphate, triethylamine, and phosphoric acid are analytically pure. The methanol of the treated samples is analytically pure, and the water is ultrapure water.

1. **Methods and Results**

**2.1 HPLC Conditions**

**2.1.1 HPLC conditions for the detection of ephedrine hydrochloride and pseudoephedrine hydrochloride**

Chromatographic column is AgilentZORBAXC18 column (5μm, 4.6 × 250mm). Acetonitrile was used as mobile phase A, and 0.1% phosphoric acid (adjusted pH to 2.6 by triethylamine) was used as mobile phase B, and gradient elution was performed as provided in table 6. Flow rate was 1ml/min. Detection wavelength was 210nm. Column temperature was 25℃ unless under special condition. The injection volume was 10μl.

Table 6: Gradient elution procedure

| Time | A(%) | B(%) |
| --- | --- | --- |
| 0-20 | 3 | 97 |
| 21-40 | 15 | 85 |
| 41-50 | 3 | 97 |

Note: A represents mobile phase A, B reprents mobile phase B

**2.1.2 HPLC conditions for the detection of Sinapine thiocyanate**

Chromatographic column is Shiseido CAPCELLPAKMGⅡC18 column (5μm, 4.6 × 250mm). The mobile phase was acetonitrile-0.08 mol/L-potassium dihydrogen phosphate solution (13:87). The flow rate was 1ml/min. Detection wavelength was 326nm. Column temperature was 30℃ unless some special conditions. The number of theoretical plates should be no less than 5000. Under this chromatographic condition, the sample and the standard separated from each other, and they could achieve baseline separation from other impurity peaks.

**2.2 Solution Preparation**

**2.2.1 Preparation of Standard Solution**

**For the detection of ephedrine hydrochloride and pseudoephedrine hydrochloride**

The appropriate amount of ephedrine hydrochloride and pseudoephedrine hydrochloride was precisely weighed, and the two standards were dissolved in methanol to acquire the mixture standard solutions at a concentration of 1mg/ml, and then diluted with mobile phase into a mixture standard solution at a concentration of 50μg/ml.

**For detection of sinapine thiocyanate**

The appropriate amount of sinapine thiocyanate standard was precisely weighed, and it was dissolved in the mobile phase to obtain the standard solution at a concentration of 50μg/ml.

**2.2.2** **Preparation of the Sample Solutions (the batch numbers of Majie cataplasm: 20130901)**

**For the detection of ephedrine hydrochloride and pseudoephedrine hydrochloride**

Took 6 pieces of Majie cataplasm, removed the coating, cut and mixed well. It was accurately weighed 1.0g and put into a 50ml round bottom flask, added precisely 20ml of 80% methanol, and was weighed again. It was extracted for 30min in a water bath at 80℃ under the reflux method. Took out, and cooled to room temperature. Weighed again, and made up the lost weight with 80% methanol. Shook well, filtered, and concentrated the filtrate (5ml) to dry under reduced pressure at 50℃. Dissolved and transferred the residue with the mobile phase to the mark in a 5ml volumetric flask. Shook well, and filtered through a 0.45μm water-based filter membrane.

**For the detection of sinapine thiocyanate**

Took 6 pieces of Majie cataplasm, removed the coating, cut and mixed well. It was accurately weighed 2.0g and put into a 50ml round bottom flask, added precisely 20ml of 80% methanol, and was weighed again. It was extracted for 1h under the reflux method. Took out, and cooled to room temperature. Weighed again, and made up the lost weight with 80% methanol. Shook well and filtered and got the further filtrate.

**2.2.3 Preparation of negative sample solution**

**The negative sample for Mahuang**

Took drugs except for Mahuang in compliance with the prescription of Majie cataplasm, handled it under the process of the extraction and the molding technique of Majie cataplasm, and then made the negative sample solution under the method mentioned in 2.2.2.

**The negative sample for Baijiezi**

Took drugs except for Baijiezi according to the prescription of Majie cataplasm, handled it under the process of the extraction and the molding technique of Majie cataplasm, and then made the negative sample solution following the method mentioned in 2.2.2.

**2.3 Detection**

The accurate injection volume of the above solutions was 10μl separately. Injected them into the liquid chromatograph, detected the peak area, and calculated by external standard method.

**3 Method validation**

**3.1 System suitability**

Prepared the sample solution and the negative sample solution according to methods mentioned in 2.2.2 and 2.2.3, and injected 10μl of the standard solution, the sample solution and the negative sample solution respectively according to the chromatographic conditions of 2.1.

The results showed that the same chromatographic peak appears at the corresponding position in the sample solution chromatogram and the standard solution chromatogram, but the negative sample solution did not detect this peak, indicating that other ingredients in the prescription did not interfere with the detection of ephedrine hydrochloride, pseudoephedrine hydrochloride and sinapine thiocyanate in the final product.

**3.2 Linearity**

54.40mg of ephedrine hydrochloride and 54.65mg of pseudoephedrine hydrochloride were accurately weighed, poured into a 50ml volumetric flask, and methanol was added to make a mixed standard solution of ephedrine hydrochloride (1.085mg/ml) and pseudoephedrine hydrochloride(1.092mg/ml). The solution was used as a stock solution. It was precisely diluted with the mobile phase, and injected according to the chromatographic conditions of 2.1.

The injection volume was 10μl, and the peak area was recorded. The linear regression of the peak area (Y) with the content (X) yielded the regression equation of ephedrine hydrochloride: Y = 2085.8X + 26.109, the correlation coefficient r = 0.9999, and the regression equation of pseudoephedrine hydrochloride: Y = 2068.1X + 26.876, the correlation coefficient r = 0.9999. The results showed that under the above chromatographic conditions, ephedrine hydrochloride had a good linear relationship in the range of 0.0217μg-5.4250μg, and pseudoephedrine hydrochloride had a good linear relationship in the range of 0.0218μg-5.4600μg.

13.29mg of sinapine thiocyanate standard was accurately weighed, poured into a 10ml volumetric flask, and the mobile phase was added to make a sinapine thiocyanate standard solution with a concentration of 1.3051mg/ml, which was used as a stock solution. It was precisely aspirated and diluted with the mobile phase, and injected according to the chromatographic conditions of 2.1. The injection volume was 10μl, and the peak area was recorded. The linear regression of the peak area (Y) with the content (X) yielded the regression equation: Y = 1480.2X-29.059, the correlation coefficient r = 0.9999. The results showed that under the above chromatographic conditions, sinapine thiocyanate had a good linear relationship in the range of 0.026μg to 6.526μg.

**3.3 Accuracy**

**3.3.1 Preparation of the standard solutions**

22.11mg ephedrine hydrochloride standard and 8.51mg pseudoephedrine hydrochloride standard were accurately weighed, poured into a 50ml volumetric flask, and methanol was added to the mark as the mixed standard solution (among ephedrine hydrochloride: 0.4409mg/ml, Pseudoephedrine hydrochloride: 0.170mg/ml).

10.19mg of sinapine thiocyanate standard was accurately weighed, poured into a 50ml volumetric flask, and the mobile phase was added to dilute to the mark as a solution of sinapine thiocyanate with a concentration of 0.2001mg/ml for reference.

**3.3.2 Recovery Rate**

Took three copies of 0.5ml, 1.0ml, and 1.5ml of the mixed standard solution respectively for a total of nine tubes. After the methanol had been naturally evaporated, added precisely 0.5g of the sample including ephedrine hydrochloride (0.087%) and pseudoephedrine hydrochloride (0.026%) to each tube, and then operated following the method mentioned in 2.2.2. The sample solution was injected according to the chromatographic conditions mentioned in 2.1, and the injection volume was 10μl for detection. The results showed that the average recoveries of this analytical method were 98.87% and 98.81%, respectively, and the RSD values were 1.49% and 1.93% (n=9).

Took three copies of 0.5ml, 1.0ml, and 1.5ml of the 0.2001mg/ml sinapine thiocyanate standard solution for a total of nine tubes. After the solvent was naturally evaporated, added precisely 1.0g of the sample (sinapine thiocyanate content is 0.021%) to each tube, and then operated following the method mentioned in 2.2.2. The sample solution was injected according to the chromatographic conditions mentioned in 2.1, and the injection volume was 10μl for detection. The result showed that the average recoveries of this analytical method was 100.49%, and the RSD values was 1.73% (n=9).

As we could see, the recovery rate was satisfactory.

**3.4 Precision**

**3.4.1 Intermediate precisiont**

A sample of Majie cataplasm was prepared by another tester at different times following the items of 2.2.2. The sample was performed on the Agilent 1260 HPLC analyzer with the chromatographic conditions mentioned in 2.1. The results of the content of Ephedrine hydrochloride and sinapine thiocyanate were basically the same. It showed that the intermediate precision of this method was good.

**3.4.2 Instrument precision**

Took the mixed standard solution of ephedrine hydrochloride (0.0542mg/ml) and pseudoephedrine hydrochloride (0.0546mg/ml), handled with the chromatographic conditions mentioned in 2.1. Injected 6 consecutive injections with a single volume of 10μl. The results of the area showed that the method had good instrument precision.

Took the standard solution of sinapine thiocyanate (0.0534mg/ml), and handled with the chromatographic conditions mentioned in 2.1. The injection volume was 10μl. Repeated the injection 6 times. Calculated the peak area, RSD=0.14% (n=6). The results showed that this method had good instrument precision.

**3.4.3 Repeatability**

Precisely took 6 samples from the same batch, handled according to the method mentioned in 2.2.2, and applied the chromatographic conditions mentioned in 2.1. The calculated RSD of ephedrine hydrochloride and pseudoephedrine hydrochloride was 2.2% (n=6), and the calculated RSD of sinapine thiocyanate was 0.9% (n=6). The results indicated that the method had good repeatability.

**3.5 Range**

Precisely weighed 0.5g, 1.0g, and 1.5g of samples, and treated them according to the method mentioned in 2.2.2. According to the chromatographic conditions mentioned in 2.1, injected 10μl of the sample solution and recorded the peak area. It could be seen from the results that the detection method could be favorable when the sample was 1.0g and the fluctuation range was 50%-150%.

**3.6 Durability**

In the durability test, the following factors that may change were examined:

**3.6.1 Columns of the same type from different brands or different batch number**

When other factors remained unchanged, the influence of the same type of columns from different brands was investigated. The results showed that although the columns of the same type from different brands affected the retention time, while the resolution of the chromatographic peaks and the results maintained unchallenged.

**3.6.2 Column temperature change**

The same sample was taken and tested at different column temperatures. The results showed that the temperature change within ±5°C influenced little on the results.

**3.6.3 Changes in the mobile phase composition ratio**

When other factors remained unchanged, the influence of slight changes in the composition ratio of the mobile phase was examined. The results demonstrated that a slight change in the composition ratio of the mobile phase exerted a significant effect on the retention time. However, there were neither considerable effects on the separation of the chromatographic peaks nor for the results.

**3.6.4 Flow rate**

When other factors remained unchanged, examined the influence of flow rate. The results showed that although the change in flow rate showed an impact on the retention time, the resolution of the chromatographic peaks and the results barely changed.

**3.6.5 Solution stability**

The sample solution was tested at 0, 2, 4, 6, 8, 12, and 24 h according to the chromatographic conditions mentioned in 2.1. The injection volume was 10μl, and the peak area was calculated. From the results, the RSDs of ephedrine hydrochloride and pseudoephedrine hydrochloride were 1.22% and 2.54% (n=7), respectively.

The sample solution was tested at 0, 2, 4, 6, 8, 12, and 24 h according to the chromatographic conditions mentioned in 2.1. The injection volume was 10μl, and the peak area was calculated. From the results, the RSD of sinapine thiocyanate was 0.93% (n=6).

The content of ephedrine hydrochloride, pseudoephedrine hydrochloride and sinapine thiocyanate showed almost no change at all, indicating that the samples were stable within 24 hours.

**4.The detection of the sample**

The results of the detection of 3 batches of Majie cataplasm are shown in table 7, 8.

Table7. The total amount of ephedrine hydrochloride and pseudoephedrine hydrochloride in 3 batches of Majie cataplasm

| Batch No. | Sampling weight(g) | Total amount of ephedrine hydrochloride and pseudoephedrine hydrochloride (%) | Average (%) | Average weight of 20 pieces(g/piece) | Total amount of ephedrine hydrochloride and pseudoephedrine hydrochloride of each piece(mg/piece) |
| --- | --- | --- | --- | --- | --- |
| 20131101 | 1.0001 | 0.106 | 0.106 | 6.89 | 7.30 |
|  | 1.0004 | 0.105 |  |  |  |
| 20131201 | 1.0107 | 0.118 | 0.117 | 7.01 | 8.20 |
|  | 0.9998 | 0.116 |  |  |  |
| 20131202 | 1.0014 | 0.112 | 0.112 | 6.54 | 7.32 |
|  | 1.0024 | 0.111 |  |  |  |

Note: The average weight of 20 pieces is weighed without the coating.

Table 8. The content of sinapine thiocyanate in 3 batches of Majie cataplasm

| Batch No. | Sampling weight(g) | Sinapine thiocyanate content (%) | Average (%) | Average weight of 20 pieces (g/patch) | Sinapine thiocyanate content of each piece (mg/ piece) |
| --- | --- | --- | --- | --- | --- |
| 20131101 | 2.0002 | 0.020 | 0.020 | 6.89 | 1.38 |
|  | 2.0127 | 0.020 |  |  |  |
| 20131201 | 2.0045 | 0.022 | 0.022 | 7.01 | 1.54 |
|  | 2.0109 | 0.023 |  |  |  |
| 20131202 | 2.0023 | 0.018 | 0.018 | 6.54 | 1.18 |
|  | 2.0019 | 0.018 |  |  |  |

Note: The average weight of 20 pieces is weighed without the coating.

**5. Content standards of ephedrine hydrochloride, pseudoephedrine hydrochloride and sinapine thiocyanate in Majie cataplasm**

From the statistical analysis of the results, it could be known that the average content of the three batches of pilot samples containing ephedrine hydrochloride and pseudoephedrine hydrochloride was about 7.6mg. Based on 70% of the average, the total amount of ephedrine hydrochloride and pseudoephedrine hydrochloride should be no less than 5.3mg.

From the statistical analysis of the results, it could be known that the average content of the three batches of pilot samples containing sinapine thiocyanate was 1.4mg. Based on 70% of the average, the content of sinapine thiocyanate should be no less than 0.98mg.

**Conclusion**: According to these experiments, the technique of Majie cataplasm is reliable. It has good reproducibility and can be mass-produced, which lays a good foundation for the subsequent medicinal mechanism experiments.

The load of each piece of Majie cataplasm is uniform. The contact area is 63cm^2^ (length 9cm x width 7cm), which is converted to a mouse application area of about 0.2cm^2^. We cut the plaster into small square patches of 0.45cm x 0.45cm. C57BL/6J mice are prepared for subsequent animal experiments.


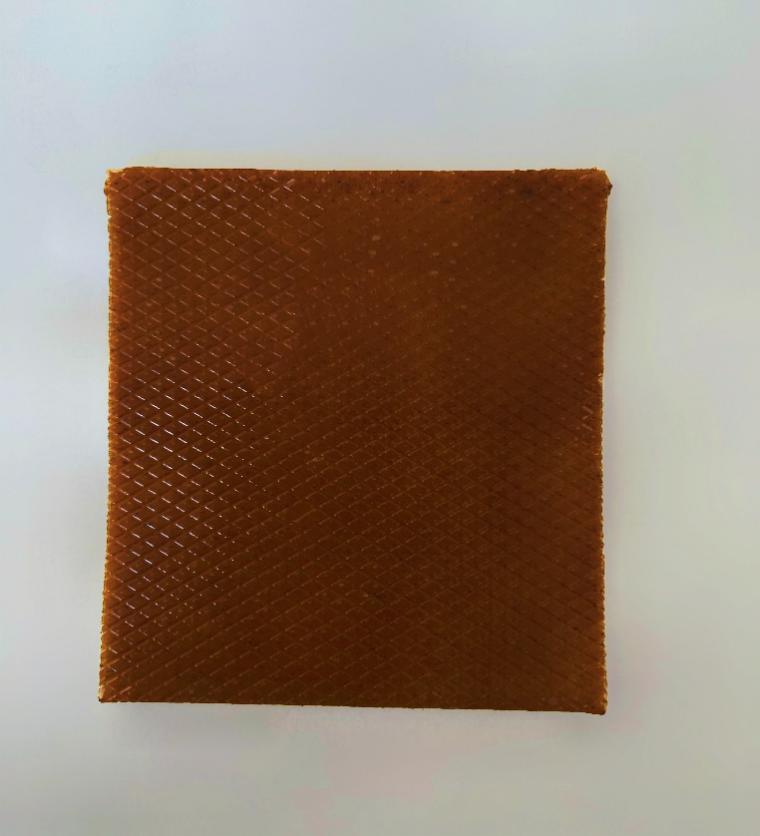


Fig 4 The final product of Majie cataplasm (length 9cm x width 7cm)

**Abbreviation**

| abbreviation | the full name |
| --- | --- |
| HPLC | High Performance Liquid Chromatography |
| TLC | Thin-Layer Chromatography |
| NP-800 | Sodium polyacrylate |
| XL-10 | Polyvinylpolypyrrolidone |
| PVP | polyvinylpyrrolidone |

**References**

[1] Xiaotong WANG, Xueqian WANG, Huihua QU, et al. Optimization of Extraction Technology for Effective Ingredients from Majie Pingchuan Cataplasma by Orthogonal Test and Transdermal Characteristic Investigation[J]. Chinese Journal of Experimental Traditional Medical Formulae. 2013,19(15):1-4.

[2] Hui Kong, Huihua Qu, Baoping Qu, et al. Correlation between the transdermal characteristics of pseudoephedrine and amygdalin in majiepingchuan in vitro[J]. Journal of Traditional Chinese Medicine. 2016, 36(2): 238-242

[3] Yuehong XU, Hui YE, Sutao GUAN, et al. Study on release and permeation behavior of Baijiezi Tufang hydrogel patch in vitro[J]. Chinese Traditional Patent Medicine. 2011(12):45-49.
